# Why Do We Move Our Head to Look at an Object in Our Peripheral Region? Lateral Viewing Interferes with Attentive Search

**DOI:** 10.1371/journal.pone.0092284

**Published:** 2014-03-19

**Authors:** Ryoichi Nakashima, Satoshi Shioiri

**Affiliations:** 1 Research Institute of Electrical Communication, Tohoku University, Sendai, Japan; 2 Core Research for Evolutional Science and Technology (CREST), Japan Science and Technology Agency, Tokyo, Japan; VU University Amsterdam, Netherlands

## Abstract

Why do we frequently fixate an object of interest presented peripherally by moving our head as well as our eyes, even when we are capable of fixating the object with an eye movement alone (lateral viewing)? Studies of eye-head coordination for gaze shifts have suggested that the degree of eye-head coupling could be determined by an unconscious weighing of the motor costs and benefits of executing a head movement. The present study investigated visual perceptual effects of head direction as an additional factor impacting on a cost-benefit organization of eye-head control. Three experiments using visual search tasks were conducted, manipulating eye direction relative to head orientation (front or lateral viewing). Results show that lateral viewing increased the time required to detect a target in a search for the letter T among letter L distractors, a serial attentive search task, but not in a search for T among letter O distractors, a parallel preattentive search task (Experiment 1). The interference could not be attributed to either a deleterious effect of lateral gaze on the accuracy of saccadic eye movements, nor to potentially problematic optical effects of binocular lateral viewing, because effect of head directions was obtained under conditions in which the task was accomplished without saccades (Experiment 2), and during monocular viewing (Experiment 3). These results suggest that a difference between the head and eye directions interferes with visual processing, and that the interference can be explained by the modulation of attention by the relative positions of the eyes and head (or head direction).

## Introduction

The visual world is highly complex, and visual experience is rich and detailed. However, our visual system cannot process all the visual information around us simultaneously. We usually focus visual attention on one object or on one local area at a time, and shift attention serially in order to scan the surrounding environment. Although the location of attentional focus and fixation position are not always the same (e.g., [1], [2]), attention is on or around a fixation position for most of the duration of visual processing. It is also the case that visual attention is usually automatically directed to the saccade goal prior to a saccadic eye movement [3]–[5].

Studies that examined eye-head coupling during saccades [6]–[13] have demonstrated that saccades made to eccentricities within the limits of the full-scale eye-in-head range (approximately ±50 degrees) may or may not be associated with a head movement. Centrifugal gaze shifts executed without a head movement result in the target being fixated with a greater eye-in-head eccentricity, while gaze shifts executed with a combined eye-head saccade usually result in a near-central final eye-in-head position. The unconscious decision of whether to move the head during a gaze shift likely depends on multiple factors, including the expected duration that gaze will be maintained in the general vicinity of the new target and an occult weighing of the costs and benefits of executing a head movement [7], [8]. Among the costs of moving the head is the energy required to accelerate and decelerate a large mass in a short time. Among the costs of not moving the head (or conversely, the benefits of moving the head and thereby reducing final eye-in-head eccentricity), fixation accuracy and stability decrease at far-eccentric eye positions [14]. However, costs and benefits cannot be based on final eye eccentricity alone. Studies on reading [15], [16] have shown that the head often moves with the eyes, even though the average book page could be scanned using eye-only saccades alone without ever exceeding the eye eccentricities at which eye movements begin to deteriorate. In everyday life, we often align the eyes and head while investigating a visual object in detail. Thus, in addition to considerations of motor control, there may be factors related to visual processing, perception, cognition, and/or attention that are factored into the hypothesized cost-benefit analysis underlying eye-head coupling.

Although no studies have reported an effect of head direction on attention, several studies have shown a relationship between head and eye movements and visual attention [17], [18]. For example, Doshi and Tridevi [17] reported that different eye and head movements were found with different attention states: the eyes moves prior to the head when attention is captured by an exogenous cue, whereas the head moves prior to the eyes when attention shifts by an endogenous cue. Further, there are studies suggesting that the eye direction relative to the head influences perception and/or cognition. Blohm and colleagues [19]–[21] reported that accuracy of depth estimation or a perceived hand position can degrade when eye and head directions differ. They suggested that visual sensory inputs are coded in different reference frames respectively (eye-centered and head-centered), and if the head and eyes are directed to different orientations, noise added to the reference frame transformation degrades spatial perception accuracy. In addition, Dunham [22] reported that observers tend to move their head toward any visual stimulus presented peripherally during tasks they consider difficult. These effects on perception/cognition may be due to general attentional modulation for performing tasks.

**Figure 1:**
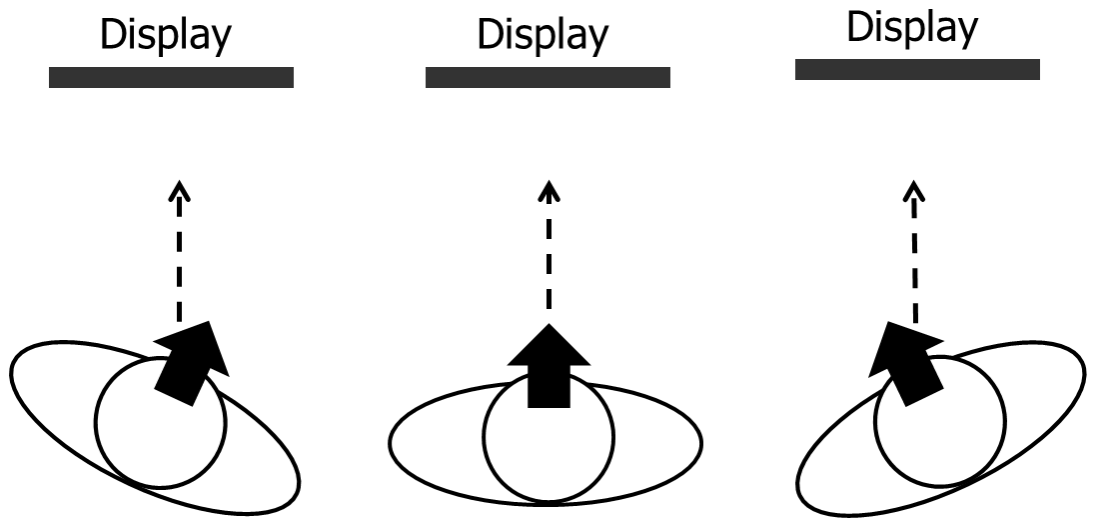
Experimental setup. Each figure represents an eye direction condition (left, front, right). Thick arrows indicate the head (and body) orientation of the participant, and dashed arrows indicate eye direction.

In the present study, we examined whether lateral viewing (i.e., the viewing condition where the eye directions are largely different from the head direction) influences perception/cognition, focusing on preattentive and attentive processing. We conducted a series of visual search experiments, manipulating an observer’s head (and body) direction relative to a stimulus display, such that the display was viewed frontally and laterally (left and right) (Figure 1). Visual search is one of the most widely used tasks to examine preattentive and attentive visual processing [23], [24]. Previous studies have identified two types of visual searches: a parallel search and a serial search (e.g., [23]–[31]). These searches can be used to investigate effects of attention on visual processing (but see [32]–[34]). In a parallel search, the target pops out and observers need not intentionally allocate attention to each item, and the number of items on the search display does not influence the performance. In a serial search, observers must allocate attention to each item sequentially and more time is required to detect the target with increased item number. Only preattentive processing is assumed to be involved in parallel search, and attentive processing is assumed to be involved in serial search in addition to the preattentive processing. Based on the assumption, we examined the effect of lateral viewing on preattentive and attentive processing in visual search. If the effect of the lateral viewing is different between a serial search and a parallel search, we would suggest that the lateral viewing influences attentive processing and/or processing after attentive selection in visual search. Alternatively, if the effect is the same for both a serial search and a parallel search, we would suggest that the lateral viewing influences preattentive processing and/or general processing.

## Experiment 1

We used two types of visual search tasks: a parallel search task and a serial search task [23]–[31]. A participant searched for a target letter T among distractor letter Os (a typical parallel search task) or among Ls (a typical serial search task). The participant reported whether the target, T, was pointing to the left or to the right. We compared the performance for T/O task and T/L task in order to examine whether lateral viewing had different effects on parallel and serial visual processing.

The critical manipulation in this and the following experiments is the directional relationship between the participant and the display (i.e., head direction), namely the front and lateral conditions. These two conditions correspond to controlling eye direction straight ahead (front) and lateral to the head (lateral). A majority of people will choose to execute a combined eye-head saccade rather than to sustain so great an eye-in-head eccentricity [10], [11], raising the possibility that 30° lateral viewing raises the costs of visual processing.

### Methods

#### Participants

The participants in Experiment 1 were 16 undergraduate and graduate students (mean age: 22.6 years; SD: 3.37 years). All participants had normal or corrected-to-normal vision. All of them reported that they could look at the stimuli via lateral viewing relatively easily. All experiments were approved by Tohoku University’s institutional review board, and written informed consent was obtained from all participants.

#### Apparatus

Presentation of the stimuli and the recordings of participants’ responses were controlled by a computer, using the Psychophysics Toolbox [35], [36]. Stimuli were displayed on a 37-inch liquid crystal display (1280×720 pixels).

#### Stimuli

**Figure 2:**
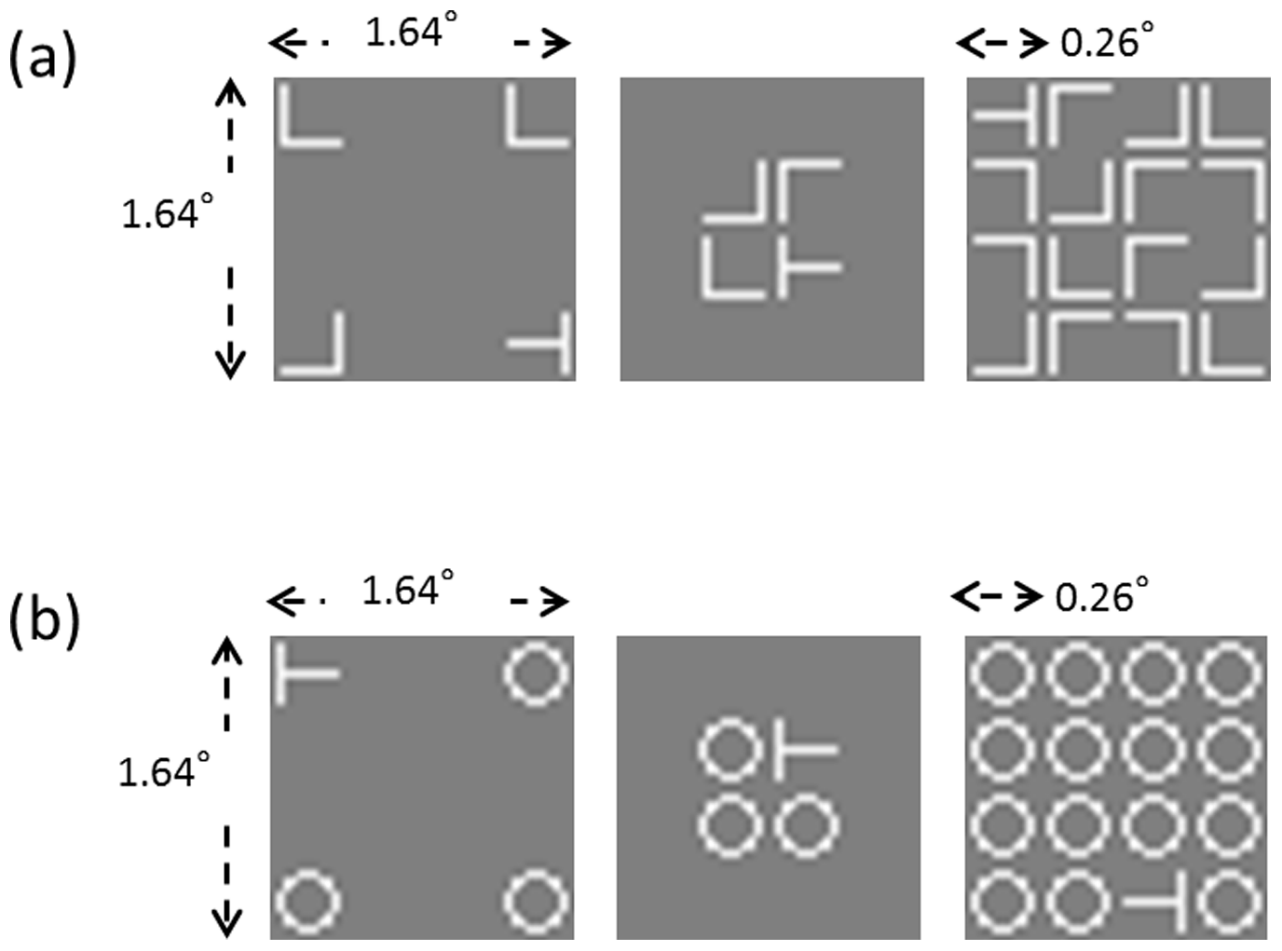
Examples of stimuli. Examples of visual search displays of (a) serial search (a spatial configuration search with T/L task) and (b) parallel search (a feature search with T/O task). There were three stimulus configuration conditions in each task: 4-corner (left), 4-center (center), and set size 16 (right).

We prepared white (325.6 cd/m^2^) T, O, and L letters drawn on a gray background (63.8 cd/m^2^) as the visual search items. The target was a T rotated 90° to the left or right of vertical. In the serial search task, distractors were Ls rotated 0°, 90°, 180°, or 270°. In the parallel search task, Os were used as distractors. Each letter subtended 0.26°×0.26° of visual angle. Stimuli were presented on an imaginary 4×4 grid, within which each cell was 0.41°×0.41° of visual angle. The whole search display was within a 1.64°×1.64° area. We used two set size conditions: 4 and 16 items. In the condition with a set size of 4, 1 target and 3 distractors were presented in the 4 corner cells (named the 4-corner condition) or 4 central cells (named the 4-center condition). In the 16 set size condition, 1 target and 15 distractors were presented in all cells (Figure 2).

#### Procedure

The experiment was conducted in a dark room. The viewing distance was 60 cm (from the center of the left and right eyes to the display), fixed by a chin and forehead rest. We manipulated the directional relationship between the participant and the display (head direction condition). In the front condition, the participant’s head was directed along the surface normal of the display. In the lateral (left or right) conditions, the participant’s head (and body) was directed 30° to the left or right of the line connecting the display and the participant (see Figure 1). The manipulation of the head direction was to control eye direction (front or lateral) relative to the head. That is, there were three eye direction conditions: front, left, and right. In each eye direction condition, the participant’s head direction was fixed by a chin and forehead rest throughout the experimental block. To change the eye direction condition (i.e., head direction condition), we rotated the chin and forehead rest between the experimental blocks.

At the beginning of each trial, a fixation cross was presented and the participant was instructed to press a button to start the trial when ready. Five hundred milliseconds after they pressed the button, the fixation cross was replaced with a visual search display (no blank display in-between) and the display remained until the participant responded. The task was to search for a T and to report its orientation (left or right) as accurately and quickly as possible by pressing a response button.

Three eye direction conditions were blocked, and the block order was randomized across participants. Each block consisted of 256 trials: 64 trials in each of the 4 conditions created by a 2 (parallel or serial search task) × 2 (set size 4 or 16) factorial format. The condition of set size 4 included 32 trials of the 4-corner and 4-center conditions. The order of trials was randomized in each block.

In the lateral viewing conditions, the participants were instructed to maintain lateral viewing throughout each trial and allowed to rest freely between the trials. They were not required to maintain lateral viewing between trials. We assume that such self-paced starts for trials minimized possible fatigue caused by maintaining lateral viewing.

### Results

We analyzed reaction time (RT), which we defined as the time between stimulus presentation and participants’ response button press. The overall accuracy was high (above 96.5% for all participants). Since RT distributions are generally skewed, we calculated a median RT for each participant from correct responses. The left and right eye direction conditions were collapsed and analyzed as the lateral condition, because there was no significant difference in RT between the two conditions, *F*s <1, *p*s >.4.

**Figure 3:**
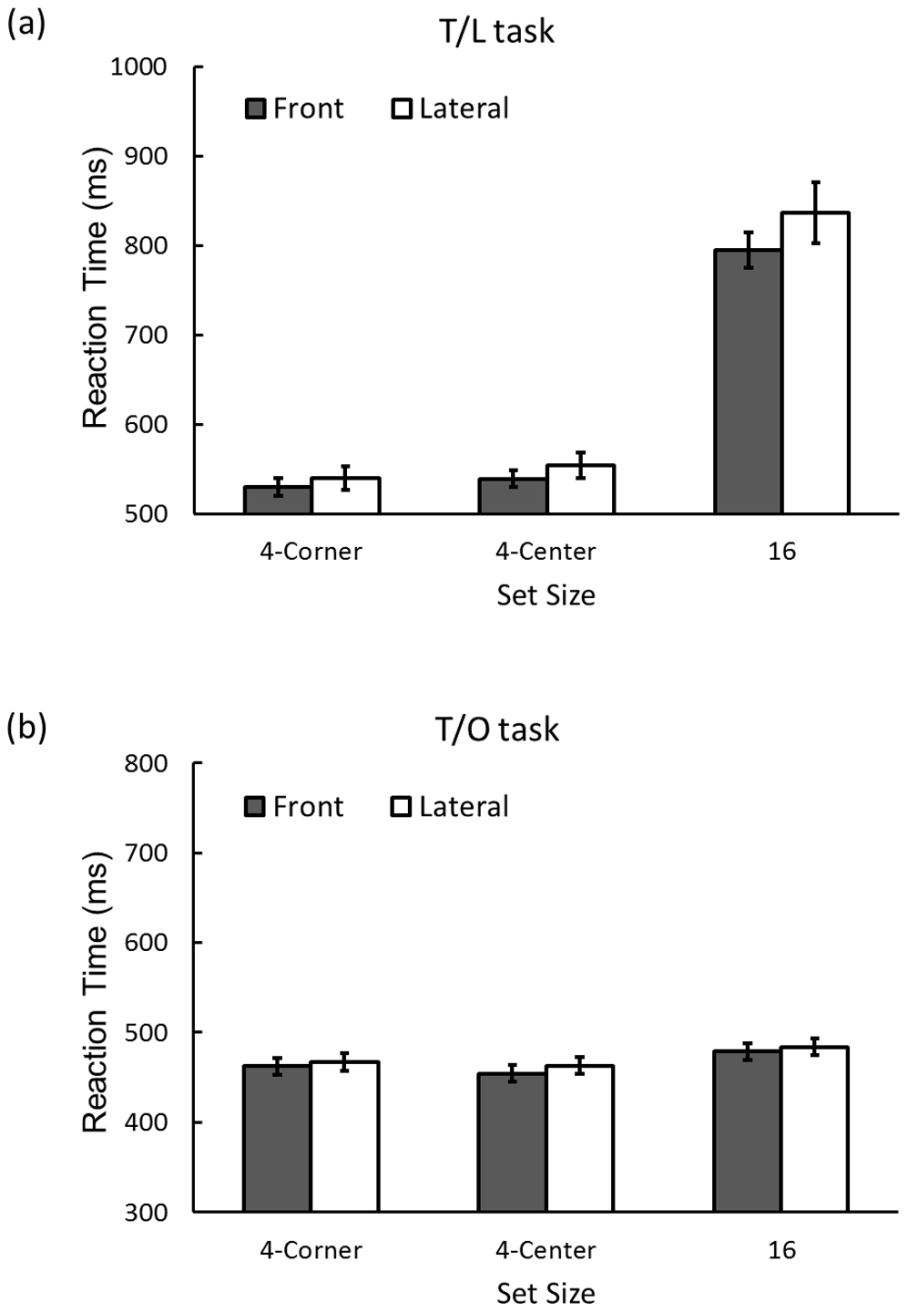
Results of Experiment 1. RT for correct-response trials by task for (a) serial search (i.e., T/L task) and (b) parallel search (i.e., T/O task) in Experiment 1. Error bars indicate standard errors.

Figure 3 shows RTs in Experiment 1. We conducted an analysis of variance (repeated measures ANOVA) on RTs with factors for eye direction (front vs. lateral), task (T/O vs. T/L), and stimulus configurations (set sizes 4-corner vs. 4-center vs. 16). There was a trend towards longer RTs when the eyes were directed to either side relative to the head (lateral viewing) than when the eyes were straight ahead, *F*(1, 15) = 3.73, *p*<.08, η_p_^2^ = .20. RTs were longer in the T/L search than in the T/O search tasks, *F*(1, 15) = 180.99, *p*<.001, η_p_^2^ = .92. Participants took a longer time to detect a target from among many items, *F*(2, 30) = 200.58, *p*<.001, η_p_^2^ = .93. The task and the set size also showed an interaction, *F*(1, 15) = 190.43, *p*<.001, η_p_^2^ = .93. RTs increased as set size became large in the T/L task, *p*<.001, but not in the T/O task, *p*>.1. Eye direction and task showed an interaction, *F*(1, 15) = 6.23, *p*<.03, η_p_^2^ = .29. RTs were longer when the eyes were oriented laterally relative to the head than when the eyes were directed straight ahead in only the T/L task, *p*<.01. The eye direction and set size also interacted, *F*(2, 30) = 4.44, *p*<.03, η_p_^2^ = .23. In the set size 16 condition, RTs were longer during lateral viewing, *p*<.001, whereas there were no differences in the set size 4 conditions, *p*s >.1. Three-way interaction was significant, *F*(2, 30) = 3.79, *p*<.04, η_p_^2^ = .21, and RT was longer in the lateral eye condition than in the front eye condition in only the T/L task with set size 16, *p*s <.001. There were no significant differences between eye direction conditions in the other tasks, *p*s >.1.

To distinguish the effect on preattentive and attentive processing further, we used the slope of the function of RT vs. item number (i.e., search function) in the T/L task where the effect of lateral viewing was clear. The slope, processing time per item, is expected to reflect attentive processing such as sequential allocation attention to each item (e.g., [23], [24], [26]). We calculated the slope of the search function of each task by dividing the difference in RTs of two set size conditions (two set size 4 conditions were collapsed) by the difference in the numbers of items. We also calculated the intercept of the function, which indicates the time required to perform the task independently of processing each stimulus item, such as the acquisition of global information and the button press (e.g., [24], [26]). The slope was significantly larger in the lateral viewing condition (24 ms/item) than in the front viewing condition (21 ms/item), *t*(15) = 2.24, *p*<.05, while the intercept was not different between these two viewing conditions (lateral viewing: 451 ms, front viewing: 448 ms), *t*(15) = .65, *p*>.5.

### Discussion

We examined the effect of eye direction (front or lateral) on visual search performance considering both parallel (i.e., preattentive) and serial (i.e., attentive) processing. Results show that performance deteriorated in the serial search during lateral viewing. In contrast, performance in the parallel search was not influenced by the eye direction. Further, the results showed the slope increased in the lateral viewing condition. In serial searches, the slope reflects the efficiency of visual processing for each item (i.e., visual attentive processing), and the intercept reflects the processing without attention, such as the acquisition of global information [24]. These results suggest that lateral viewing especially interferes with higher stages of visual processing including the attention control process in serial visual search.

To rule out the possibility that fatigue caused by maintaining lateral viewing interfered with visual search, i.e., only searches with long RTs might have been influenced by lateral viewing because of the fatigue independently of attention control, we conducted an additional experiment where participants maintained lateral viewing for a certain period of time before starting the search (pre-observation). In this experiment, T/O and T/L tasks with a set size of 4 were used for 9 participants, manipulating pre-observation time. Participants were asked to fix their gaze on a fixation point presented either 500 ms (short) or 1000 ms (long) before stimulus presentation. In the 1000 ms fixation presentation condition (i.e., long pre-observation), participants were forced to maintain lateral viewing for 500 ms longer than in the same task in Experiment 1. Therefore, based on the results in Experiment 1, the time for maintaining lateral viewing could be about 1550 ms (about 550 ms of RT plus 1000 ms of pre-observation time) in the long pre-observation condition. This is longer than the time for maintaining lateral viewing in the T/L task with the set size of 16 (about 1350 ms), which consisted of 500 ms of presentation fixation plus about 850 ms of RT in Experiment 1. If fatigue with long lateral viewing duration interfered with visual search, RTs for long pre-observation in lateral viewing condition would be longer than in front viewing condition even in a set size of 4 conditions. We found that, for the T/L task, RTs in the front and lateral viewing conditions were 538 ms and 536 ms for short pre-observation and 534 ms and 539 ms for long pre-observation, respectively. For the T/O task, RTs in the front and lateral viewing conditions were 471 ms and 467 ms for short pre-observation and 467 ms and 469 ms for long pre-observation, respectively. The differences in pre-observation time had little or no effect on visual search RT, *F*s <1. Thus, fatigue or any other effects caused by maintaining lateral viewing for as long as 1000 ms do not explain the difference between front and lateral viewing conditions found in Experiment 1.

The results imply that the state of visual attention could change depending on eye direction relative to the head. Attentive visual search may be interrupted when a viewer looks at an object of interest without directing the head to the object. However, there remain other possibilities that could account for the lengthened RTs during lateral viewing. We examined two alternative interpretations: (1) The efficiency of eye movement may be reduced when eyes are directed toward side regions, or (2) the incoming visual information in each eye becomes different when viewers look toward the side, and that difference may increase the time required to process visual information. We examined these possibilities in the following experiments.

## Experiment 2

Eyes may move most efficiently and/or with less restriction when the eyes are directed in the same direction as the head, that is, when looking straight ahead. In fact, the accuracy of eye movement control declines as a function of a target eccentricity [14]. Although we used stimuli presented within the fovea (1.64°×1.64° of visual angle), this does not mean that the eyes did not move at all when performing the search tasks. We cannot, therefore, exclude the possibility that eye movement control was impaired during lateral viewing.

In Experiment 2, we examined whether lateral viewing would interfere with search performance under conditions where no saccadic eye movement occurred while searching for targets. The visual search display was presented briefly (for 100 ms). Saccadic eye movements could not occur during such brief presentation because the latency of saccade is no shorter than 100 ms except for special cases (e.g., [37]–[39]). It should be noted that a target can be searched serially even without eye movements, even after the visual stimuli vanished [40], [41]. Although attentional shift and eye movement usually correlate (overt attention), visual attention can shift independently of eye movement (covert attention) (e.g., [1], [2]). An important role of covert attention in visual search has been shown in the results of a serial search without eye movement [41].

### Methods

We used the serial search task (i.e., T/L task) with a set size of 16, where the effect of lateral viewing was expected to be strong. The participants in Experiment 2 were 8 graduate students (mean age: 23.8 years; SD 2.71 years). All of them had participated in Experiment 1. The apparatus and stimuli were identical to those in Experiment 1.

The procedure was the same as in Experiment 1 except that the stimulus was presented for a fixed duration of 100 ms. After the termination of the stimulus display, a blank display of a gray field (63.8 cd/m^2^) was presented until the participant responded. Three eye direction conditions (front, left, and right) were blocked (2 blocks for front and 1 each for left and right), and the block order was randomized across participants. Each block consisted of 96 trials.

### Results and Discussion

Because of the shorter presentation duration, accuracies were lower than those of Experiment 1. Percentages of correct responses were 88.5% for the front condition and 86.5% for the lateral condition. There was no significant difference between the accuracies in the front and lateral eye direction conditions, *t*(7) = .92, *p*>.3.

We analyzed the RT data of trials with correct responses. RT for the lateral condition (828±54 ms) was longer than for the front condition (779±69 ms), *t*(7) = 2.50, *p*<.05, replicating the result that lateral viewing interfered with visual search. This indicates that lateral viewing may interfere with the serial allocation of attention in the representation of the stimulus in the visual brain (e.g., [42]). Since there was not enough time to move the eyes, longer RT (i.e., worse performance) in the lateral viewing condition cannot be attributed to any effect of eye movements.

## Experiment 3

Retinal images from the left and right eyes are usually different, and there is a specific binocular difference when the eyes are directed laterally, namely size difference. The size difference between the two retinal images in the lateral viewing in Experiments 1 and 2 was 4.9% or 0.08° in visual angle. The size difference is much larger than both visual and stereo acuities, and such a difference could impair stereopsis [43]. It may, therefore, impair search performance as well. To examine the effect of retinal image differences between the two eyes, we repeated the visual search experiment with one eye. In addition, we compared visual search performances between the binocular (Experiment 2) and monocular viewing conditions.

We used three different viewing distances in the monocular condition. Since we defined the viewing distance as the distance from the midpoint between the left and right eyes to the display in Experiments 1 and 2, distance from the display differed slightly between the two eyes, which caused difference in retinal size. If pupillary distance were 6 cm, the difference in viewing distance between the left and right eyes would be roughly 3 cm (5% of the viewing distance). In this experiment, we used viewing distances of 57, 60, and 63 cm to examine the potential influence of viewing distance.

### Methods

The participants in Experiment 3 were 8 graduate students (mean age: 23.7 years; SD: 2.72 years), of whom 5 participated in both Experiments 1 and 2, and 2 participated in Experiment 1. The apparatus and stimuli were identical to those in the previous experiments. We used the serial search task with a set size of 16, which was presented for 100 ms.

Viewing eye, eye direction, and viewing distance conditions were blocked. In the left (right) eye condition, the participant’s right (left) eye was covered with an eye patch. The experimental procedure was the same as that of Experiment 2. Eighteen conditions, created by 2 (left or right eye) × 3 (front, left, or right) × 3 (57, 60, or 63 cm of viewing distance), were blocked. Each block consisted of 64 trials. Half of the participants conducted the experiments with their right eye first, and the remaining participants with left eye first. The block order was randomized across participants.

### Results and Discussion

Because of the shorter presentation duration, accuracies were lower relative to Experiment 1. Percentages of correct responses were about 78.8% under the experimental conditions and there was no significant difference between any of the conditions, *F*s <1.8, *p*s >.1. We analyzed the RT data of trials with correct responses. In the analysis of this experiment, we did not collapse the left and right eye direction conditions, because we were interested in examining the relationship between the viewing eye and its direction.

**Figure 4:**
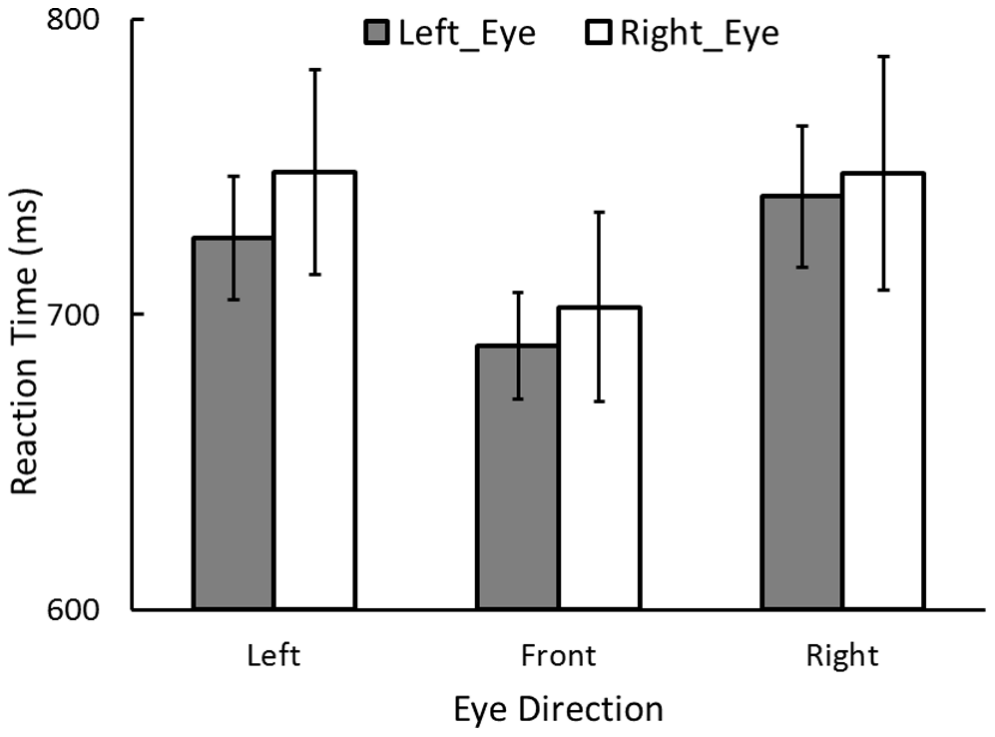
Results of Experiment 3. RT for correct-response trials by eye direction and eye condition in Experiment 3, collapsed over the viewing distance conditions. Error bars indicate standard errors.

Figure 4 shows RTs in this experiment collapsed over the viewing distance conditions. We conducted a repeated measures ANOVA on RTs with factors for viewing eye (right, left), eye direction (front vs. right vs. left), and viewing distance (57 vs. 60 vs. 63 cm). The main effect of eye direction was significant, *F*(2, 14) = 8.72, *p*<.01, η_p_^2^ = .55. The RT was shorter when an eye was gazing straight ahead than when the eye was oriented to either side relative to the head, *p*s <.01. The difference between the left and right eye direction conditions was not significant, *p*>.8. Since lateral viewing effect was found with monocular viewing, we suggest that the impairment in search performance that occurs with lateral viewing cannot be attributed to the size difference in visual information input.

The main effect of viewing distance and its interactions were not significant, *F*s <2, *p*s >.1. We conclude, therefore, that small distance variation is not a critical factor in the present experiments, and it is not related to the impairment in search performance that occurs with lateral viewing.

The main effect of viewing eye and the interaction between the eye and its direction were not significant, *F*s <2.2, *p*s >.1. Visual search performance did not depend on viewing with either eye and showed no particular effect of eye-direction combinations. Visual search performance was not influenced by whether the eyes were directed inward (i.e., toward the nose) or outward (i.e., toward the ears).

We also examined the effect of dominant eye, classifying the RT into dominant eye and non-dominant eye. Each participant’s dominant eye in the front viewing was determined by the “Dominant Eye Test Card” downloaded freely from USAEyes (http://www.usaeyes.org/lasik/library/Dominant-Eye-Test.pdf). The dominant eye of five participants was the left, and that of three participants was the right when tested front viewing. When participants looked toward the left the dominant eye of all participants was left, and when they looked toward the right it was right for all participants (see [44]). The main effect of the eye (dominant or non-dominant) and the interactions were not significant, *F*s <1.1, *p*s >.3. No effect of the dominant eye on visual search performance was found.

We compared the lateral viewing effect between the monocular and binocular viewing conditions to examine whether there is any additional effect in binocular viewing. We compared percentage increase in RT due to lateral viewing between binocular viewing (Experiment 2) and monocular viewing (Experiment 3). We compared the data of 5 participants who participated in both experiments. In the monocular viewing condition, only a 60 cm viewing distance condition was used, and eye conditions were collapsed for this analysis. Results showed a speed-accuracy tradeoff: mean accuracy was higher in binocular viewing (85.1%) than in monocular viewing (78.6%), whereas RT was longer in binocular viewing (843 ms) than in monocular viewing (707 ms). We controlled for this by combining RT and accuracy in a single search index, dividing RT by the mean accuracy for each participant in each condition (e.g., [45], [46], see also [47]). The search indexes were 0.89±0.04 (front) and 0.97±0.08 (lateral) in binocular viewing, and 0.85±0.03 (front) and 0.91±0.05 (lateral) in monocular viewing. Percentage increase of the indexes were not significantly different between binocular viewing (7.7%) and monocular viewing (7.9%), *t*(4) = .05, *p*>.9. Even when the data was analyzed with Experiment (i.e., binocular vs. monocular viewing) as a between-participants factor (the data of all participants were analyzed), there was no significant difference between between binocular viewing (8.1%) and monocular viewing (8.5%), *t*(14) = .09, *p*>.9. This suggests that the difference between monocular and binocular vision does not influence the lateral viewing effect decisively.

## General Discussion

Several studies have suggested that whether the head moves or not is based on evaluation of cost and benefit in the controlling process of head and eye movements [7]–[9]. The present study found an impact of the relative positions of the eyes and head on cognitive efficiency. As such, cognitive efficiency may represent an additional factor that is weighed in the cost-benefit analysis proposed to underlie the variable coupling of head and eye movements. In Experiment 1, we conducted a parallel search (T/O task) and a serial search (T/L task) experiments, where the participant’s eye direction was the same or different from that of head direction (front vs. lateral). The RT was significantly longer when participants directed their eyes laterally than when they directed their eyes straight ahead in the serial attention-demanding search task, but not (or minimally) in the parallel preattentive (i.e., non-attention-demanding) search task. These results suggest that the difference between the head and eye directions interferes with attentive processing in visual searches. Experiments 2 and 3 ruled out alternative interpretations. Experiment 2 ruled out the possibility that impairment of eye movement control lengthened RT in lateral viewing conditions. Experiment 3 ruled out the possibility that the size difference of two retinal images during lateral viewing impaired visual search performance.

The effect of lateral viewing on visual search performance was not accounted for by any of the simple optical factors we explored (e.g., eye movement, binocular vs. monocular viewing, direction with respect to viewing eye). We, therefore, suggest that there is attention modulation by the eye direction relative to the head (or head direction) for visual attentive processing in visual search.

Assuming attentional modulation for visual processing, there are two possible interpretations for the influence of head direction, which are not exclusive. First, the impairment due to lateral viewing can be attributed to the reduction of the localization accuracy of attentional focus. That is, the accuracy to localize attentional focus may decrease when the head and eye directions are different, as in a similar sense for spatial perception in a localization task where the participant judged the relative location of a visual stimulus presented around the hand, which was not visible ([21] see also [19], [20]). Burns et al. suggested that noise arising from the misalignment of the reference frames for the eyes and head degrades accuracy of hand location perception, resulting in more uncertainty with than without eye and head misalignment. The same noise may also reduce efficiency of visual search in the present experiments, and the reduction effect may be on the attentional process because only serial search was influenced.

Second, with the misalignment of the reference frames for the eyes and head, attention can be directed not only to a fixation position but also to a head direction, and visual performance is higher when the head is directed to the visual stimuli than when it is not. Attentional resources may get divided between head direction and eye direction when they are different, perhaps with a larger weight for eye direction, and processing efficiency may be better when the eyes and head directions are same. This may be related to the fact that observers tend to move their head toward visual stimuli presented peripherally during tasks they feel difficult [22]. To perform a difficult task, they may want to focus their attentional resources by aligning the orientations of the eyes and head.

Our suggestion in this study is that lateral viewing can interfere with serial attentive processing in visual search. However, this is not the exclusive and definitive explanation for the effect of lateral viewing on visual perception. Although previous studies suggested that whether attentive processing is involved in or not is the major difference between the parallel and serial searches (e.g., [23], [24]), the task difficulty is also different between these two tasks. Thus, one alternative explanation is that lateral viewing interferes with general perception and the effect may become clearer when the task is relatively difficult. At least, our results suggest that lateral viewing does not influence all perceptual processing. Yet, most of the issues about lateral viewing remain unclear. To understand the effect of lateral viewing on visual perception and cognition fully, the effect of lateral viewing on attention and/or visual perception should be examined with the other visual tasks. These are important challenges in the future research.

In conclusion, we suggest that the attentional processes involved in serial visual search are modulated by the relative directions of the eyes and head. An examination of the degree to which the head and eyes are aligned during specific daily visual tasks may provide important insights into the control mechanisms underlying the direction and division of attentional resources.
